# Tetrahedral Framework Nucleic Acid Relieves Sepsis‐Induced Intestinal Injury by Regulating M2 Macrophages

**DOI:** 10.1111/cpr.13803

**Published:** 2025-01-22

**Authors:** Tingting Tan, Jiajie Li, Wensi Fan, Kangni Shang, Chujun Yang, Xiaohao Liu, Shihui Zhu, Tong Liu, Junjie Wang, Yingchuan Li, Yunfeng Lin

**Affiliations:** ^1^ Department of Critical Care Medicine, Shanghai Tenth People's Hospital Tongji University School of Medicine Shanghai People's Republic of China; ^2^ Key Laboratory of Pathogen‐Host Interaction Ministry of Education Beijing People's Republic of China; ^3^ School of Basic Medicine and Clinical Pharmacy China Pharmaceutical University Nanjing People's Republic of China; ^4^ State Key Laboratory of Oral Diseases, National Clinical Research Center for Oral Diseases, West China Hospital of Stomatology Sichuan University, Chengdu, Department of Burns and Plastic Surgery Chengdu People's Republic of China; ^5^ Shanghai Children's Medical Center Shanghai Jiao Tong University School of Medicine Shanghai People's Republic of China; ^6^ Department of Critical Care Medicine, Zhongshan Hospital Fudan University Shanghai People's Republic of China

## Abstract

This study aimed to clarify the role and mechanism of tetrahedral framework nucleic acids (tFNAs) in regulating M2 macrophages to reduce intestinal injury. An intestinal injury model was established by intraperitoneal injection of lipopolysaccharides (LPS) in mice to explore the alleviating effects of tFNAs on intestinal injury. Inflammatory factors were detected by quantitative polymerase chain reaction (qPCR) and enzyme‐linked immunosorbent assay (ELISA). The intestinal barrier and permeability were assessed using western blotting and immunohistochemistry. Macrophages in the gut were localised and quantified using immunofluorescence. Western blotting was used to investigate the role and mechanism of tFNAs in regulating macrophages and alleviating inflammation in the injured intestines. These results show that tFNAs attenuated sepsis‐induced intestinal injury. tFNAs can also promote the intestinal barrier reconstruction and reduce intestinal permeability. In vivo, tFNAs accelerated the aggregation of M2 macrophages at an early stage of injury and reduced the number of M1 macrophages in the intestine. In addition, tFNAs enhanced the clearance ability of intestinal macrophages. They activated the signalling and transcription activating factor 1(STAT1) and cytokine signalling inhibitory factor 1/3 (SOCS1/3) pathways by increasing the expression of the phagocytic receptor Mertk. These findings indicated that tFNAs can alleviate sepsis‐induced intestinal injury by regulating M2 macrophages, providing a new option for treating intestinal injury.

## Graphical abstract

Mechanism of tFNAs activating Mertk/STAT1/SOCS signalling pathways and regulating M1/M2 macrophages polarisation in the intestine. tFNAs promoted the M2 macrophages polarisation and reduced the M1 macrophages polarisation in the environment of sepsis‐induced intestinal injury, thereby reducing the release of inflammatory factors, alleviating intestinal injury and repairing the intestinal barrier.

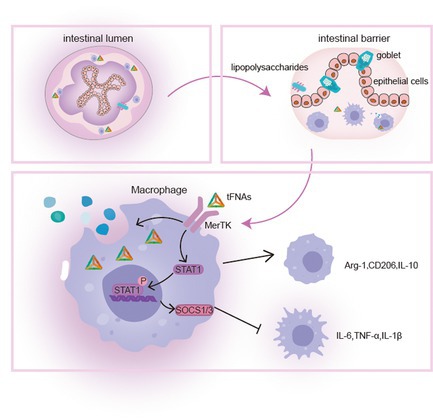

## 1 Introduction

Sepsis is a life‐threatening multi‐organ dysfunction caused by the host's abnormal response to infection [1]. The intestine is the most commonly affected organ in sepsis. It is the initiating organ for multi‐organ dysfunction [1]. The interaction between intestinal cytokine storm and barrier damage jointly promotes sepsis development. Epidemiological data shows that the incidence of intestinal injury in patients with sepsis can be as high as 90%, and it is widespread in the elderly, immunocompromised and patients with underlying intestinal diseases [2, 3, 4]. The current treatment strategies for sepsis‐induced intestinal injury include supportive therapy, anti‐infection therapy and immunomodulation [5, 6]. However, drug resistance caused by antibiotic abuse limits the effectiveness of antibiotic treatment. Patients with sepsis also face difficulties in implementing enteral nutrition [7, 8]. There is an urgent need to develop specific treatment plans for sepsis‐induced intestinal injury to suppress intestinal inflammatory responses and repair intestinal barriers. Macrophages are gatekeepers of intestinal immune homeostasis. Macrophages clear apoptotic cells and cell debris at the injury site through phagocytosis, preventing the aggravation of the injury [9]. Classical M1 macrophages secrete inflammatory factors that amplify the inflammatory response; M2 macrophages secrete anti‐inflammatory cytokines that promote tissue damage repair [10]. Therefore, enhancing macrophage efferocytosis and promoting M2 macrophage polarisation may be beneficial for the clearance of intestinal inflammation [11].

Mertk is a member of the Tyro‐Axl‐Mer (TAM) receptor tyrosine kinase family and is mainly expressed on the surface of macrophages [12]. As a transmembrane receptor, Mertk mediates phagocytosis by macrophages. By binding with ligand Protein S 1 (PROS1), it recognises the phosphatidylserine signal expressed on the surface of apoptotic cells, promoting the clearance effect of macrophages on apoptotic cells [13]. p‐Mertk is involved in the activation of Mertk and regulates STAT1/SOCS pathways [14]. Mertk activation stimulates macrophage polarisation towards the M2 type [15]. Wu et al. confirmed that in a traumatic brain injury model, Mertk could regulate the polarisation of microglia/macrophages towards the M2 type [16]. A small amount of research has confirmed the expression of Mertk in the intestinal tissue [17, 18]. However, there is currently no evidence to prove that Mertk expression in the intestine regulates inflammation and affects macrophage polarisation.

tFNAs are stable tetrahedral nanoparticles assembled from four specific single‐stranded DNA fragments [19, 20]. Its advantages, such as strong stability, high tissue permeability and programmability, have attracted widespread attention in biomedical research [21, 22, 23, 24]. As nucleic acid drugs, tFNAs regulate innate and adaptive immunity [25, 26, 27]. Previous studies suggested that tFNAs facilitate the polarisation of macrophages and transformation into the M2 phenotype [28]. tFNAs have a strong tissue penetration ability [29]. Still, it is not clear whether they can accumulate at an effective concentration in the intestine. Further exploration is required to determine whether tFNAs can protect the intestine by regulating macrophages. Hence, in this study, the sepsis‐induced intestinal injury was simulated by intraperitoneal injection of LPS to explore the role of tFNAs in regulating intestinal macrophages, alleviating inflammation, and repairing the intestinal barrier.

## 2 Materials and Methods

### 2.1 Synthesis and Characterisation of tFNAs

Based on the base‐pairing principle, tFNAs nanomaterials were self‐assembled from four equimolar amounts (1 μm) of single‐stranded sequence‐specific DNA (ssDNA) fragments in TM buffer solution (composed of 50 mM MgCl_2_ and 10 mm trishcl at pH 8.0). The synthesised tFNAs were stored in a refrigerator at 4°C for future use.

### 2.2 Cell Culture and Cellular Uptake of tFNAs

The RAW 264.7 cells were acquired from the American Type Culture Collection (Bethesda, MD, USA). To confirm the internalisation of tFNAs by RAW 264.7 cells, we treated tFNAs and ssDNA with cyanine5 (Cy5). Following the separate coculturing of the tFNAs with RAW 264.7 cells, the fluorescence microscope (Nikon, Japan) was used to capture images.

### 2.3 Real‐Time Quantitative Polymerase Chain Reaction (RTq‐PCR)

Total RNA was extracted from mouse intestinal tissue using an RNA extraction reagent from Vazyme (Nanjing, China). The purity and concentration of RNA were measured using a nanodrop, and the RNA was diluted to 500 ng/uL with enzyme‐free water. The extracted RNA was reversed into cDNA using a reverse transcription reagent from Vazyme and stored at −20°C for future use. The PCR reaction was performed using ChamQ Universal SYBR qPCR Master Mix reagent on a Thermo Fisher 384 PCR system. The relative quantification was carried out using the 2^−ΔΔCt^ method. The primer sequences are listed in Table 1.

**TABLE 1:** Primer sequence.

Gene	Base sequence (5′‐3′)
IL‐6	F:TAGTCCTTCCTACCCCAATTTCC R:TTGGTCCTTAGCCACTCCTTC
IL‐1β	F:GCAACTGTTCCTGAACTCAACT R:ATCTTTTGGGGTCCGTCAACT
TNF‐α	F:CCCTCACACTCAGATCATCTTCT R:GCTACGACGTGGGCTACAG
Arg‐1	F:CTCCAAGCCAAAGTCCTTAGAG R:GGAGCTGTCATTAGGGACATCA
β‐Actin	F:TATGCTCTCCCTCACGCCATCC R:GTCACGCACGATTTCCCTCTCAG

### 2.4 Enzyme‐Linked Immunosorbent Assay (ELISA)

Blood was collected from the eyeball of each mouse before the mice were euthanized. After incubation at room temperature for 2 h and centrifugation at 2500 g for 20 min, the supernatant (serum) was collected. The levels of inflammatory factors IL‐6, TNF‐α and IL‐1β in serum were measured using an ELISA kit (Mlbio).

### 2.5 Western Blotting

Mouse intestinal tissue was ground using a freeze‐grinder, and total protein was extracted using RIPA (Beyotime). The protein concentration was measured using a BCA kit (Beyotime) and diluted to a uniform concentration. The primary antibody was incubated overnight at 4°C, and the secondary antibody at room temperature for 1 h. The samples were washed thrice with Tris‐buffered saline with Tween (TBST) solution between each step for 10 min. Enhanced chemiluminescence detection (ECL) was used to detect protein bands, and the results were semi‐quantitatively analysed using ImageJ software.

### 2.6 Mouse Model of Intestinal Injury: Experimental Animals and Treatment

The experimental mice used in this study were male C57BL/6J mice aged 8–10 weeks (20–22 g). The feeding conditions for mice were specific‐pathogen‐free (SPF) level, and drinking water and feed for experimental animals were provided by the Medical Science and Technology Innovation Center of Shanghai Tenth People's Hospital, all of which meet the standards for rodent feeding. The animal room was kept in a 12‐h alternating dark/light cycle, and the mice could freely eat and drink.

Sepsis‐induced intestinal injury was simulated by intraperitoneal injection of LPS (Sigma‐Aldrich, St. Louis, MO, USA) into the mice, which were then randomly divided into four groups: control group (healthy mice, intraperitoneal injection of PBS, tail vein injection of physiological saline), LPS group (model mice, intraperitoneal injection of 5 mg/kg LPS, tail vein injection of physiological saline), LPS + tFNAs group (intraperitoneal injection of 5 mg/kg LPS, tail vein injection of 250 nM tFNAs before LPS injection), and tFNAs group (intraperitoneal injection of PBS, tail vein injection of 250 nM tFNAs before PBS injection). Six hours after successful model construction, the mice were euthanized and tissue and blood samples were collected for subsequent experiments.

Inhibitors (UNC2250) dissolved in PBS were used in mice before any intervention, 5 mg/kg was injected intraperitoneally and the follow‐up intervention was started 6 h later.

### 2.7 Histological Analysis

Intestinal tissue specimens were fixed with 4% paraformaldehyde, dehydrated, embedded in paraffin, sectioned and stained with haematoxylin and eosin (HE). The histological sections were viewed and imaged using a microscope.

### 2.8 Immunohistochemistry and Immunofluorescence

The tissue sections were incubated overnight with anti‐CD206, anti‐CD86, anti‐F4/80 and anti‐MUC‐2 antibodies. The antibodies were purchased from Wuhan Service Bio‐Technology (Wuhan, China). After washing with PBS, the sections were incubated with secondary antibodies for 1 h. Sections were stained with DAPI for 10 min. Lastly, the sections were viewed and imaged under a fluorescence microscope.

### 2.9 Chemicals and Reagents

The antibody for ZO‐1 was from Wuhan Servicebio; the antibody for occludin was from Abcam; the antibodies for Mer, STAT1, p‐STAT1, SOCS1 and SOCS3 were from Cell Signalling Technology; the antibody for p‐Mer (Tyr749) was from Thermo Fisher Scientific; and UNC2250 was purchased from MCE.

### 2.10 Public Data Sources

The public scRNA‐seq datasets were downloaded from the Gene Expression Omnibus database [30], with accession numbers GSE266493. Seurat package (v3.2) in the R environment (v4.0.3) was used to screen variable features, unsupervised clustering and marker gene identification. First, in each sample, genes detected in less than 5 cells were filtered out from downstream analysis, and then NormalizeData and FindVariableFeatures functions were performed. FindIntegrationAnchors and IntegrateData functions were used to emerge data from two groups (CLP and Sham) as an integrated data assay. Cell clusters were identified via FindNeighbors and FindClusters functions with the top 10 principal components (resolution = 0.2). Clusters were annotated according to the CellMarker database, and marker genes of specific cell types were reported in the research.

## 3 Results

### 3.1 Synthesis and Characterisation of tFNAs

**FIGURE 1:**
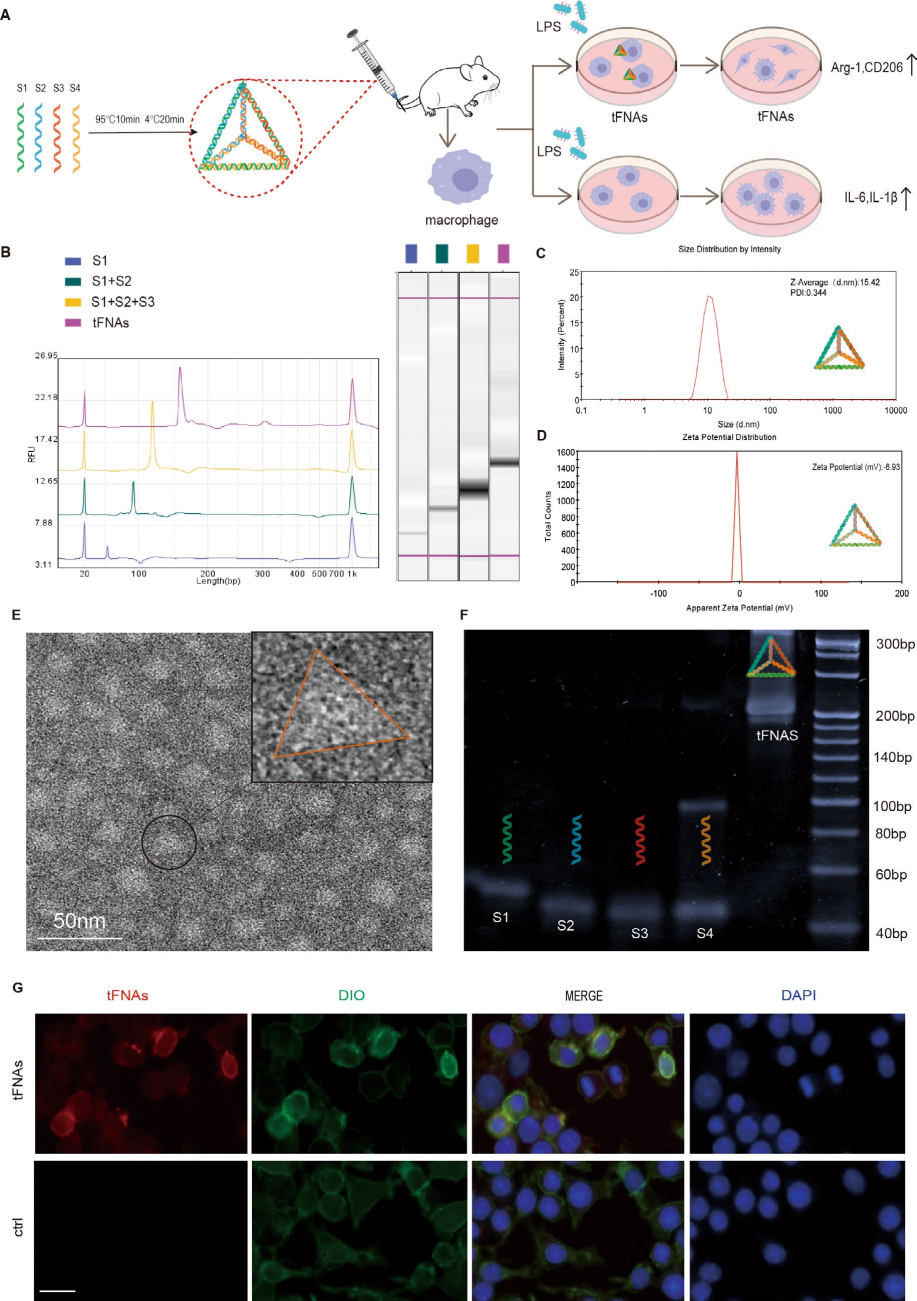
Production and characterisation of tFNAs. (A) Schematic of the structure and function of tFNAs. (B) Capillary electrophoresis results demonstrate successful preparation of the tFNAs. (C) Size distribution results for the tFNAs measured by dynamic light scattering. (D) Zeta potential results for the tFNAs were measured by dynamic light scattering. (E) TEM image of the tFNAs. (Scale bar: 50 nm) (F) The results of polyacrylamide gel electrophoresis confirmed the successful synthesis of tFNAs. (G) Immunofluorescent images to monitor the uptake of tFNAs by RAW264.7 cells. Cytomembrane was stained with DIO (green), red fluorescence was added during material preparation, and nuclei were stained with DAPI (blue)—scale bars: 50 μm.

tFNAs are stable tetrahedral structures formed by self‐assembling four single DNA strands through PCR reaction (Table 2). Following the method described in Section 2.1, four ssDNA fragments composed of specific nucleic acid sequences were added to the TM buffer solution for self‐assembly into a stable tetrahedral structure (Figure 1A). Successful synthesis of tFNAs was confirmed using capillary electrophoresis (Figure 1B). DLS measurements showed that the average size of the tFNAs particles was 15.42 nm, and the average zeta potential was −6.93 mV (Figure 1C,D). Transmission electron microscopy (TEM) revealed the tetrahedral‐like structure of tFNAs (Figure 1E). The molecular weight of the tFNAs was approximately 200 bp, as determined through PAGE (Figure 1F). To determine the cellular internalisation of tFNAs, Cy5‐modified tFNAs and ssDNA were used for immunofluorescence staining. The results showed that RAW 264.7 cells could take up tFNAs, but ssDNA was difficult to internalise (Figure 1G). tFNAs have the ability to regulate macrophage function. Classically activated M1 and significantly elevated proinflammatory cytokines were observed in macrophages stimulated by LPS. After 250 nm tFNAs pretreatment, macrophages were activated into reparative M2 under inflammatory conditions (Figure 1A).

**TABLE 2:** Base sequence.

ssDNAs	Base sequence (5′‐3′)
S1	ATTTATCACCCGCCATAGTAGACGTATCACCAGGCAGTTGAGACGAACATTCCTAAGTCTGAA
S2	ACATGCGAGGGTCCAATACCGACGATTACAGCTTGCTACACGATTCAGACTTAGGAATGTTCG
S3	CTACTATGGCGGGTGATAAAACGTGTAGCAAGCTGTAATCGACGGGAAGAGCATGCCCATCC
S4	ACGGTATTGGACCCTCGCATGACTCAACTGCCTGGTGATACGAGGATGGGCATGCTCTTCCCG
Cy5‐S1	Cy5ACGGTATTGGACCCTCGCATGACTCAACTGCCTGGTGATACGAGGATGGGCATGCTCTTCCCG

### 3.2 tFNAs Relieved Intestinal Inflammation and Restored Intestinal Barrier

**FIGURE 2:**
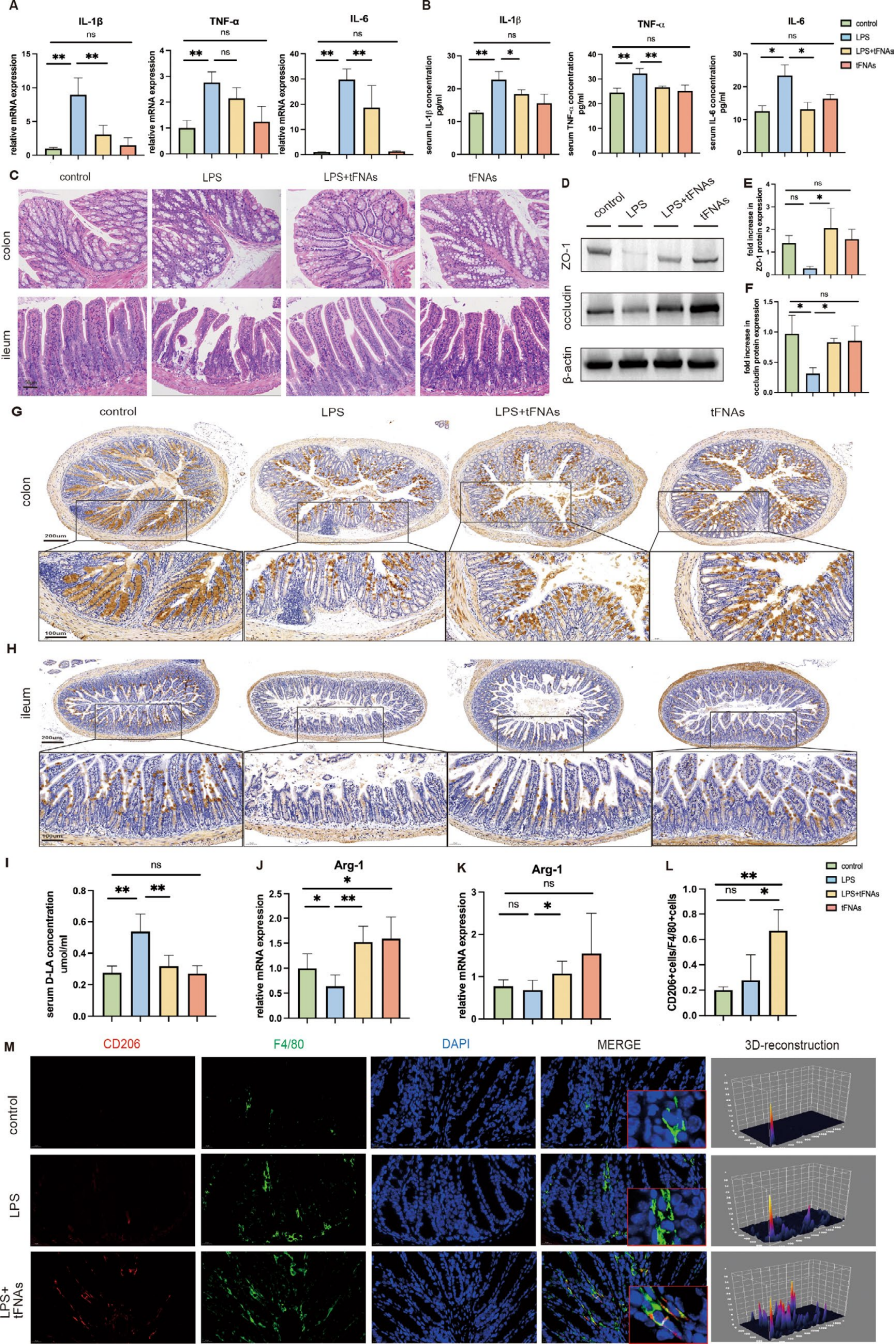
Relieved intestinal injury and increased M2 macrophages after tFNAs treatment. (A) The mRNA level of IL‐1β, TNF‐α and IL‐6 in colon. (B) Enzyme‐linked immunosorbent assay detection of IL‐1β, TNF‐α and IL‐6 in the serum. (C) H&E staining images of the ileum and colon (scale bar: 50 μm). (D–F) Western blotting results of ZO‐1(E) and occludin(F) expression level. (G, H) MUC‐2 expression in colon (G) and ileum (H) was revealed by immunohistochemical staining (IHC). (scale bar: 200 μm, scale bar: 100 μm). (I) The content of D‐lactic acid in serum. (J, K) The mRNA level of Arg‐1 in the colon and ileum. (L) Quantification showing the percentage of CD206 and F4/80 double‐positive cells. (M) Representative images of immunofluorescent staining for CD206 (red), F4/80 (green) and DAPI (blue) in the colon. Scale bar = 20 μm. Data were represented as mean ± SD, *p < 0.05, **p < 0.01.

Intestinal injury was induced by intraperitoneal injection of LPS. The experimental setup is shown in Figure S1. Samples were collected 6 h after LPS injection because the levels of inflammatory factors were highest after 6 h [31]. The results of the intestinal injury experiment (described in the Methods section) showed that the relative expression levels of IL‐6, IL‐1β and TNF‐α mRNA in the LPS + tFNAs group were suppressed compared with the LPS group (Figure 2A). Correspondingly, the serum concentration of inflammatory factors, namely IL‐6, IL‐1β and TNF‐α, were significantly decreased in the LPS + tFNAs group compared with the levels found in the LPS group (Figure 2B). Intraperitoneal injection of LPS caused structural damage and inflammatory cell infiltration in the colon and ileal tissues. Histopathological examination revealed that the intestinal tissues of the LPS group had severe mucosal damage, disordered arrangement, apparent congestion and edema compared with the intestinal tissues of the control group. tFNAs therapy significantly reduced tissue damage in the colon and ileum (Figure 2C). These results confirmed that tFNAs treatment alleviated sepsis‐induced intestinal inflammation and tissue damage.

The destruction of the intestinal barrier accompanies intestinal inflammation. To verify the effect of tFNAs on the intestinal barrier, protein expression levels of tight junctions in the colon were measured. The results showed that the expression levels of ZO‐1 and occludin were significantly lower in the LPS group than in the control group. The expression levels of tight junction proteins returned to almost normal levels after pretreatment with tFNAs (Figure 2D–F). The chemical barrier, composed of chemicals secreted by the gastrointestinal tract, is one of the four significant intestinal barriers [32]. Mucin‐2 (MUC‐2) is the main component of the intestinal mucus layer [33]. The expression of MUC‐2 in the colon and ileum was assessed through immunohistochemistry. Compared to the control group, the content of MUC‐2 in the colon of the LPS group was significantly reduced, whereas MUC‐2 was abundant after treatment with tFNAs (Figure 2G). The ileum exhibited similar results (Figure 2H). Thus, tFNAs treatment promoted the secretion of MUC‐2 and repaired the intestinal chemical barrier. D‐lactic acid is an organic acid with a lower content in the normal intestine; an increase in D‐lactate content indicates increased intestinal permeability [34]. The concentration of D‐lactate in the serum of the LPS group was significantly increased compared to the corresponding level in the control group, and tFNAs intervention restored D‐lactate to normal levels (Figure 2I). Overall, tFNAs treatment alleviated sepsis‐induced intestinal inflammation and repaired intestinal physical and chemical barriers.

### 3.3 tFNAs Promoted the Polarisation of M2 Macrophages in the Injured Intestine in the Early Stage of Inflammation

Given that tFNAs generally play an important role in mediating macrophage polarisation, we investigated whether tFNAs promote M2 macrophage polarisation in the intestine. To investigate the regulatory effects of tFNAs on intestinal macrophages, we evaluated the polarisation of intestinal M1/M2 macrophages after tFNAs intervention. The RT‐qPCR results showed that the gene expression level of Arg‐1 in M2 macrophages in the colon and ileum tissues decreased after LPS stimulation. The expression of Arg‐1 significantly increased after tFNAs treatment (Figure 2J,K). Macrophages in the colon tissue were labelled with F4/80, and M1 and M2 macrophages were labelled with CD86 and CD206, respectively. The results showed that LPS‐induced intestinal injury increased macrophages in the colon tissue compared to the control group. This is likely because sepsis‐induced intestinal injury causes the recruitment of macrophages to the colonic submucosa. The percentage of M2 macrophages was significantly increased in the LPS + tFNAs group, whereas there was scarcely any expression of M2 macrophages in the LPS group (Figure 2L,M). In addition, the LPS group showed abundant M1 macrophages, whereas the LPS + tFNAs group had relatively fewer M1 macrophages (Figure S2). In summary, tFNAs were found to regulate the polarisation of macrophages in the submucosal layer of the colon tissue, promote the aggregation of repair‐type M2 macrophages, and reduce the aggregation of pro‐inflammatory M1 macrophages.

### 3.4 Intestinal Macrophages in Sepsis Have an Excessive Inflammatory Response and Defective Regulatory Capacity

**FIGURE 3:**
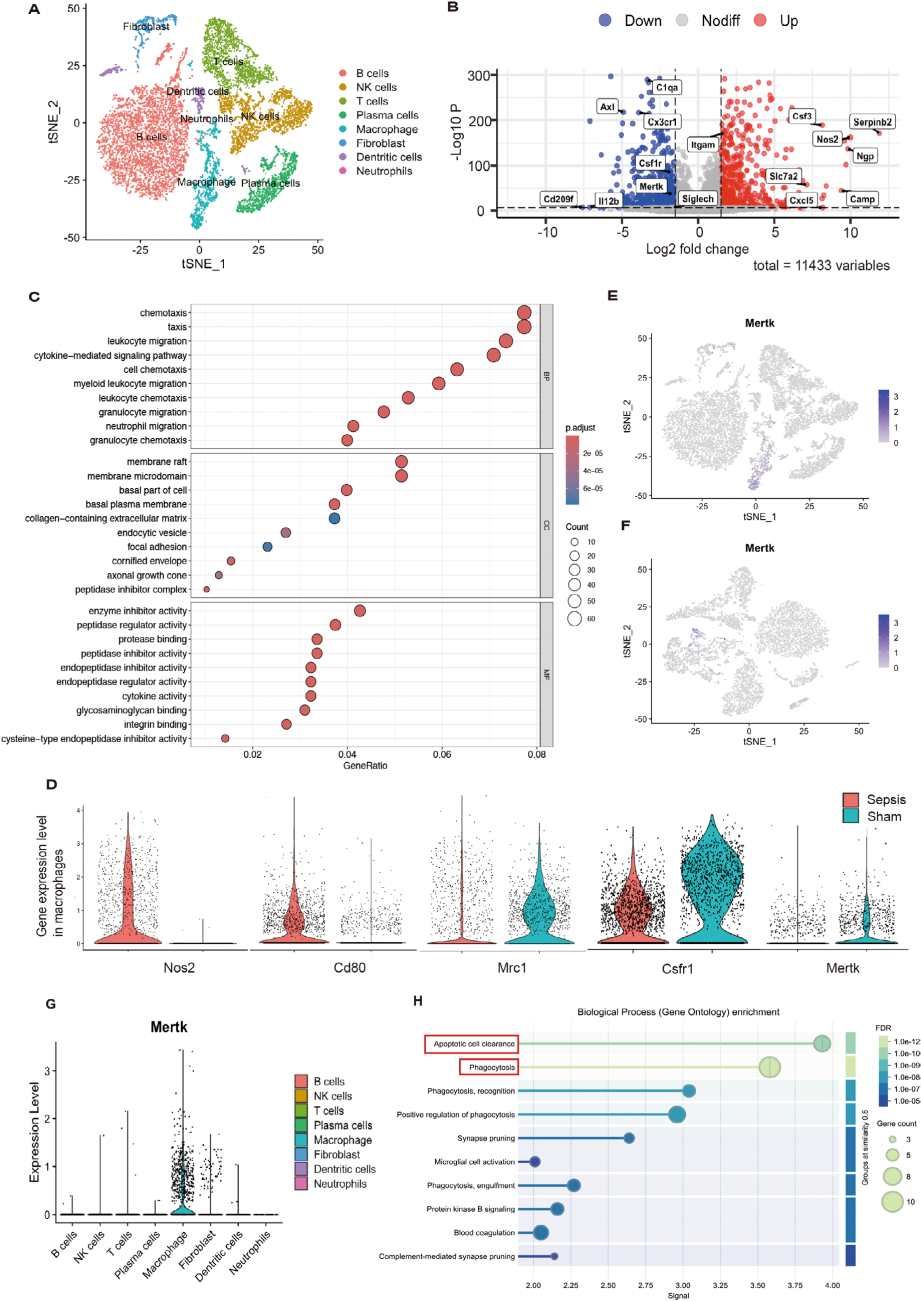
Excessive inflammatory response and defective Mertk in intestinal macrophages were observed in sepsis. (A) tSNE plot of RNA samples obtained from CD45^+^ single cells of the intestine in sepsis. Eight different clusters were identified. (B) A volcano plot of the significant DEGs between the sham and sepsis intestinal macrophages is shown. (C) The bubble plot shows both groups' significantly upregulated Gene Ontology (go) terms enriched by macrophages. (D) Violin plots showed the gene expression differences between the two groups of macrophages. (E, F) Feature plot showed that Mertk is mainly expressed in macrophage subsets in both sepsis and sham. (G) Mertk is scarcely expressed in other immune cells. (H) Functional enrichment of Mertk gene.

To better understand changes in intestinal macrophages after sepsis, we assessed single‐cell sequencing on CD45+ immune cells from the small intestine of septic mice. The t‐distributed Stochastic Neighbour Embedding (tSNE) method was used for non‐linear dimension reduction, and 8 cell clusters were identified (Figure 3A). Macrophages from sepsis and sham groups were extracted and analysed for differential genes. A total of 1705 macrophages in the sepsis group and 1302 in the sham group were acquired after quality control and filtering. After normalising the microarray data, 11,433 DEGs were detected. Volcano plot and bubble plot show significant upregulation of proinflammatory and chemotaxis‐related genes in the sepsis group compared to a sham group (Figure 3B,C), consistent with our previous findings. We analysed the expression of M1 and M2‐related genes in these two groups of macrophages. The results showed that M1‐related genes were significantly up‐regulated compared to the sham group, and M2‐associated genes were significantly downregulated in the sepsis group (Figure 3D). Among them, we observed that the expression of phagocytic receptor Mertk decreased in the sepsis group. Mertk, a receptor tyrosine kinase family member, is primarily expressed in macrophages in sepsis and sham groups (Figure 3E–G). According to GO functional enrichment, the essential functions of Mertk were enriched in the clearance of apoptotic cells and phagocytosis. This result is quoted from the STRING website (Figure 3H). Mertk can also monitor macrophage polarisation by regulating the transcription factor STAT/SOCS. The above results suggest intestinal macrophages in sepsis have excessive inflammatory response and defective clearance and regulation ability. Based on the above findings, we speculate that tFNAs can promote M2 macrophage polarisation to alleviate intestinal inflammation by regulating Mertk. Although Axl is downregulated in sepsis intestinal macrophages, it mainly regulates intracellular signal transduction, cell proliferation and differentiation (Figure S3).

### 3.5 tFNAs Activated the Phagocytic Receptor Mertk and STAT1/SOCS Pathway in the Intestine

**FIGURE 4:**
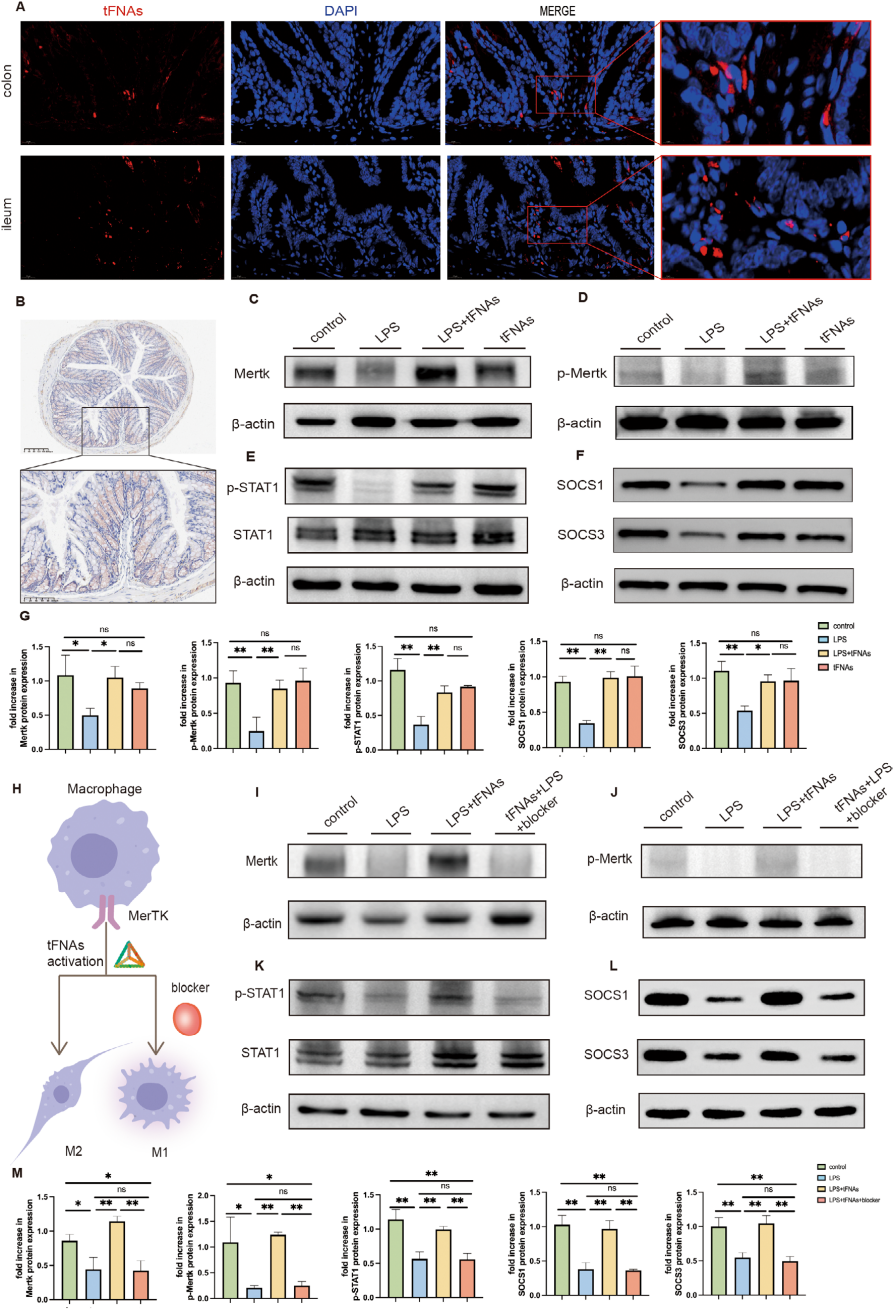
Treatment of tFNAs activated the Mertk signal pathway. (A) Representative images of immunofluorescent staining for tFNAs (red) and DAPI (blue) in the colon and ileum (Scale bar = 20 μm). (B) Immunohistochemical staining of Mertk in colon (scale bar: 100 μm). (C–F) Western blotting detection of Mertk, p‐Mertk, STAT1, p‐STAT1, SOCS1, SOCS3 expression in the colon. (G) Quantitative analysis of the protein expression levels. (H) Schematic diagram of the inhibition of Mertk altering macrophage activation by tFNAs. (I–L) Western blotting detection of Mertk, p‐Mertk, STAT1, p‐STAT1, SOCS1, SOCS3 expression in the colon. (M) Quantitative analysis of the protein expression levels. Data were represented as mean ± SD, *p < 0.05, **p < 0.01.

To better clarify the connection between tFNAs and Mertk, Cy5 fluorescence was used to label tFNAs, and the distribution of fluorescence in the intestine was detected. The results showed that tFNAs were mainly distributed in the intestinal submucosa (Figure 4A). We measured Mertk expression in the intestine using an immunohistochemistry stain. (Figure 4B). To clarify the effect of tFNAs on Mertk expression in the intestine, the level of Mertk expression in colon tissue was assessed through western blotting. Mertk expression was lower in the LPS group than in the control group. Activation of pattern‐recognition receptors with LPS in macrophages induces Mertk cleavage at the Pro^485^ proline site and shedding of the Mertk extracellular domain [12]. In the present study, we found that intervention with tFNAs significantly increased Mertk expression (Figure 4C). Mertk activation is related to the phosphorylation of the tyrosine residue 749 in the kinase domain. Accordingly, increased phosphorylation of Mertk was detected in the LPS + tFNAs group compared to that in the LPS group (Figure 4D). Mertk is also a multifunctional regulatory factor that regulates inflammatory responses by regulating multiple downstream signalling pathways, such as the STAT1/SOCS signalling pathway [12]. To elucidate the mechanism by which tFNAs regulate the inflammatory response, the expression of STAT1 and its phosphorylated protein p‐STAT1 and SOCS1/SOCS3 were measured. The results showed that p‐STAT1, SOCS1 and SOCS3 expression levels decreased after sepsis‐induced intestinal injury compared to the control group. More importantly, p‐STAT1, SOCS1 and SOCS3 expression levels significantly increased after tFNAs intervention (Figure 4E–G). These results demonstrated that treatment with tFNAs upregulated Mertk and activated it via phosphorylation. Phosphorylated Mertk activated downstream STAT1/SOCS signalling pathway, essential for inflammation regulation and macrophage polarisation. Together, these results suggested that Mertk may regulate M1/M2 polarisation by tFNAs in sepsis‐induced intestinal injury and that the STAT1/SOCS signalling pathway may mediate this effect.

### 3.6 Mertk Inhibition Reduced Activation of Mertk/STAT1/SOCS Signalling Pathway

To demonstrate that Mertk is a critical factor in the protective effects of tFNAs against intestinal injury, small‐molecule inhibitors were used to inhibit Mertk receptors in mice (Figure 4H). The activation of phosphorylated Mertk and the downstream STAT1/SOCS pathway were examined after Mertk inhibition. Deficient levels of Mertk and p‐Mertk were detected in the intestine when Mertk was inhibited (Figure 4I,J). p‐Mertk expression was barely detectable in the intestine following Mertk inhibition. This weakens the ability of tFNAs to alleviate intestinal damage. The activation of STAT1 was also tested, and the results showed that the expression of p‐STAT1 was significantly reduced in the Mertk inhibition group. A corresponding decrease in SOCS1 and SOCS3 expression was also observed (Figure 4K–M). These results demonstrated that the p‐Mertk and STAT1/SOCS signalling pathways were also inhibited when Mertk was inhibited. This may be one reason for the increased inflammation and reduced M2 macrophages.

The above results demonstrate that Mertk inhibition affects the activation of downstream STAT1 and SOCS pathways and blocks the protective effect of tFNAs by upregulating Mertk. Mertk is a crucial receptor for tFNAs that prevents intestinal injury caused by an inflammatory response by activating the Mertk and STAT1/SOCS signalling pathways.

### 3.7 The Inhibition of Mertk Suppressed the Therapeutic Effect of tFNAs

**FIGURE 5:**
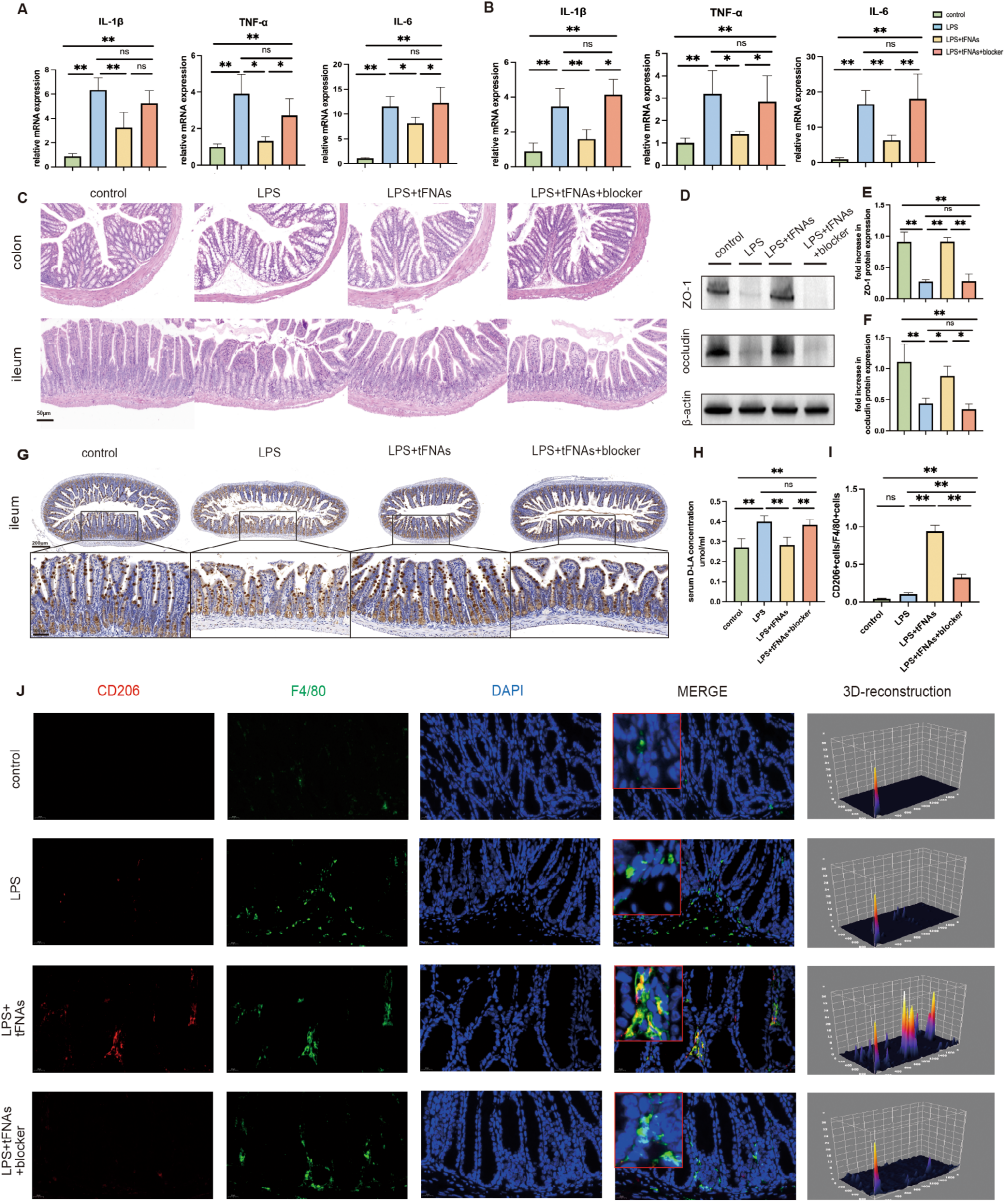
Mertk inhibition suppressed the therapeutic effect of tFNAs. (A, B) The mRNA level of IL‐1β, TNF‐α and IL‐6 in colon and ileum. (C) H&E staining images of the ileum and colon (scale bar: 50 μm). (D–F) Western blotting results of ZO‐1(E) and occludin(F) expression level. (G) MUC‐2 expression in the ileum was revealed by immunohistochemical staining (IHC). (scale bar: 200 μm, scale bar: 100 μm). (H) The content of D‐lactic acid in serum. (I) Quantification showing the percentage of CD206 and F4/80 double‐positive cells. (J) Representative images of immunofluorescent staining for CD206 (red), F4/80 (green) and DAPI (blue) in the colon. Scale bar = 20 μm. Data were represented as mean ± SD, *p < 0.05, **p < 0.01.

We hypothesized that Mertk inhibition could suppress the effect of tFNAs in alleviating inflammation and repairing intestinal barriers. After using the Mertk inhibitor, the levels of inflammatory factors in the colon and ileum tissues significantly increased compared with those in the LPS + tFNAs group, and they were similar to those in the LPS group (Figure 5A,B). HE staining images of colon and ileum tissues revealed that the LPS + tFNAs group showed a significant reduction in tissue damage compared to the LPS group, and the Mertk inhibitor reversed this effect (Figure 5C). The protein expression levels of tight junction proteins in intestinal tissues were also detected, and the results showed that the expression levels of ZO‐1 and occludin were significantly reduced after blocking Mertk, compared to the LPS + tFNAs group (Figure 5D–F). Moreover, the content of MUC‐2 after the Mertk blockade was less abundant after tFNAs treatment in both the colon and ileum (Figure S4, and Figure 5G). Serum D‐lactate content was also significantly increased in the Mertk inhibition group (Figure 5H). The above results demonstrated that Mertk inhibition abolished the role of tFNAs in alleviating intestinal inflammation and barrier repair and that Mertk was a critical factor in the protective effect of tFNAs against intestinal damage.

### 3.8 Mertk Inhibition Reduced the Polarisation of M2 Macrophages in the Intestine

To investigate the effects of Mertk on macrophage polarisation, the distribution of M1 and M2 macrophages in the intestine was evaluated after Mertk inhibition. The PCR results showed a decrease in Arg‐1 in the Mertk inhibition group (Figure S5). The immunofluorescence results showed that intervention with tFNAs reduced the aggregation of M1 macrophages in the colon tissue due to intestinal injury, while the number of M2 macrophages increased. When the Mertk is blocked, the ability of tFNAs to regulate macrophage polarisation is reversed. In the Mertk inhibition group, a significant increase in M1 macrophages and a decrease in M2 macrophages were observed, and this change was significant (Figure 5I,J, Figures S6 and S7). A similar phenomenon was also observed in the ileum tissue. Still, due to the lower abundance of macrophages in the cross‐section of the ileum tissue compared to the colon tissue, the total number of macrophages and M1/M2 subtype macrophages that could be observed was relatively small. Therefore, colonic macrophages were used for the statistical analysis and classification.

The above results demonstrated that if the Mertk receptor on the surface of macrophages is inhibited, a decrease in M2 macrophages in the intestinal tissue will likely result, leading to a severe inflammatory response. We speculated that this was due to impaired efferocytosis and that macrophages may not be able to phagocytose and clear apoptotic cells and cannot be transformed into M2 macrophages that promote repair.

## 4 Discussion

The results of the experimental study we report here confirm that tFNAs can alleviate early intestinal inflammation and barrier damage induced by LPS. After LPS activates TLR4, it inhibits Mertk expression through downstream signalling pathways [15]. The inhibition of Mertk expression has a deleterious effect on macrophages in that it may negatively affect their phagocytic and clearance abilities towards pathogens, thereby exacerbating the infection [35]. Studies have shown that the loss of Mertk signalling in the nervous system activates downstream pathways and regulates macrophage polarisation, thereby aggravating brain injury [16]. We first confirmed the suppression of LPS on Mertk expression in intestinal tissues and found that tFNAs could alleviate this effect. In conclusion, our results demonstrated that tFNAs can alleviate LPS‐induced early intestinal inflammation and repair the damaged intestinal barrier. tFNAs increased Mertk expression in the intestine, activated the downstream STAT1/SOCS pathway, reduced M1 macrophages in the intestinal tissue and promoted M2 macrophage aggregation, alleviating inflammation. These results suggested that the prophylactic use of tFNAs may help to reduce the severity of intestinal damage in sepsis and prevent further disease development. Although we believe Mertk is mainly expressed in macrophages, we cannot rule out the possibility that other immune cells show trace expression. Whether tFNAs regulate Mertk receptors expressed on the surface of different immune cells and their function requires further investigation.

The intestine, one of the organs with the most significant contact with the external environment, plays a unique role in sepsis. The intestine is not only a place for nutrient absorption but also a substantial immune organ that contains approximately 70% of the body's immune cells [36]. The treatment of sepsis is challenging, and whether immunotherapy plays a significant role in the treatment process remains controversial. We expect immunotherapy to be a breakthrough in the treatment of sepsis. Macrophages and T cells are the most abundant immune cells in the intestine [36]. As the first line of innate immunity defence, macrophages quickly activate and initiate immune responses upon pathogen detection, presenting information to other immune cells, releasing cytokines and chemicals, and engulfing and clearing pathogens [9]. Macrophages can also help maintain the integrity of the intestinal barrier and interact with other immune cells, such as T cells, to maintain intestinal immune homeostasis [37]. Given the abundance of macrophages and their early response to pathogens, we aimed to control inflammation and damage in the early stages of the disease by regulating macrophages. Previous studies demonstrated that tFNAs regulate the nervous system's microglial polarisation from M1 to M2 [28]. Li et al. demonstrated that tFNAs can promote macrophage polarisation towards M2 under LPS stimulation in cellular experiments [38]. This finding provides the theoretical basis for our hypotheses. However, in recent years, there has been a controversy over whether M1 and M2 macrophages can transform into each other [39, 40]. M1/M2 does not necessarily have such a clear boundary in diseases. They cannot be considered as two completely different groups of cells but can be used to judge the function of the cells. Macrophages regulate themselves to maintain balance, which requires consideration of the complexity of the internal environment [41]. Further in vitro and in vivo studies are needed to understand the regulation of macrophage polarisation and the interactions between signals. In the present study, we found that tFNAs increased the accumulation of M2 macrophages in the intestine and decreased the number of M1 macrophages in the early stages of injury. However, we did not distinguish between tissue‐resident macrophages and circulating macrophages. We only confirmed the regulatory effects and potential mechanisms of tFNAs on intestinal macrophages.

cGAS recognises foreign DNA entering cells and activates the STING pathway, activating the innate immune response [42]. Interestingly, as a multifunctional nucleic acid, tFNAs do not cause an excessive inflammatory response but promote inflammation repair. Our study suggests that this anti‐inflammatory effect is mediated by macrophage regulation. Previous studies have shown that functional nucleic acids act on antigen‐presenting cells and then activate T cells, promoting a connection between innate immunity and adaptive immune system [43]. Functional nucleic acids exhibit strong programmability [44, 45, 46, 47, 48, 49]. Using these functions and the characteristics of functional nucleic acids to regulate the antigen‐presenting cells and achieve immunotherapy is a very promising strategy.

## 5 Conclusion

Our study showed that the novel nanomaterial tFNAs could upregulate the Mertk receptor and activate the downstream STAT1/SOCS pathway to regulate the inflammatory response. tFNAs promoted the polarisation of macrophages to M2. They reduced the polarisation to M1 in the environment of sepsis‐induced intestinal injury, thereby reducing the release of inflammatory factors, alleviating intestinal injury and repairing the intestinal barrier. This is the first study to report the therapeutic effect of tFNAs on intestinal injury, suggesting that these nanomaterials are promising protective agents for preventing intestinal injury.

## Author Contributions

Tingting Tan, Jiajie Li and Wensi Fan conducted most of the experiments. Kangni Shang performed the western blotting and conducted experiments involving animals with the assistance of Tingting Tan. Jiajie Li, Shihui Zhu, and Tong Liu provided the essential substances. Chujun Yang and Tong Liu assisted in analysing data. Xiaohao Liu and Chujun Yang assisted in revising the manuscript. The project was led by Yunfeng Lin, Yingchuan Li, Junjie Wang and Tong Liu. Tingting Tan wrote the paper with assistance from all the authors.

## Ethics Statement

All animal experiments were approved by the Shanghai Tenth People's Hospital Medical Ethical Committee.

## Conflicts of Interest

The authors declare no conflicts of interest.

## Supporting information


Data S1.

## Data Availability

The data supporting this study's findings are available from the corresponding author upon reasonable request.
