# Supplementary material for: Tetrahedral Framework Nucleic Acid Relieves Sepsis‐Induced Intestinal Injury by Regulating M2 Macrophages
Source: Cell Prolif. 2025 Jan 22;58(5):e13803. doi: 10.1111/cpr.13803 (PMC12099223; doi:10.1111/cpr.13803)
Supplement: Supplementary file 1 — Data S1. [file CPR-58-e13803-s001.docx]

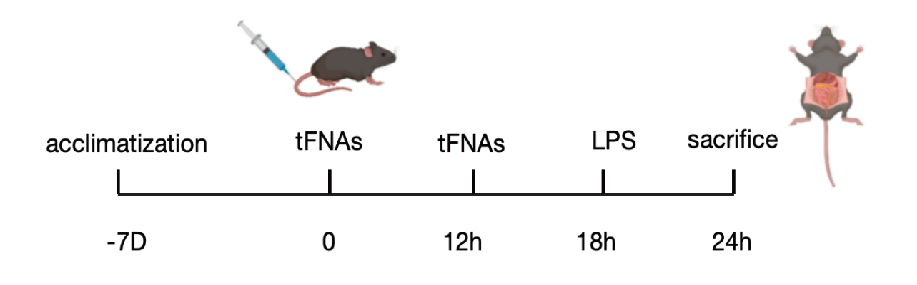


Figure S1. Schematic diagram outlining pre-treatment with the tFNAs and the establishment of LPS induced intestinal injury (n=6) .


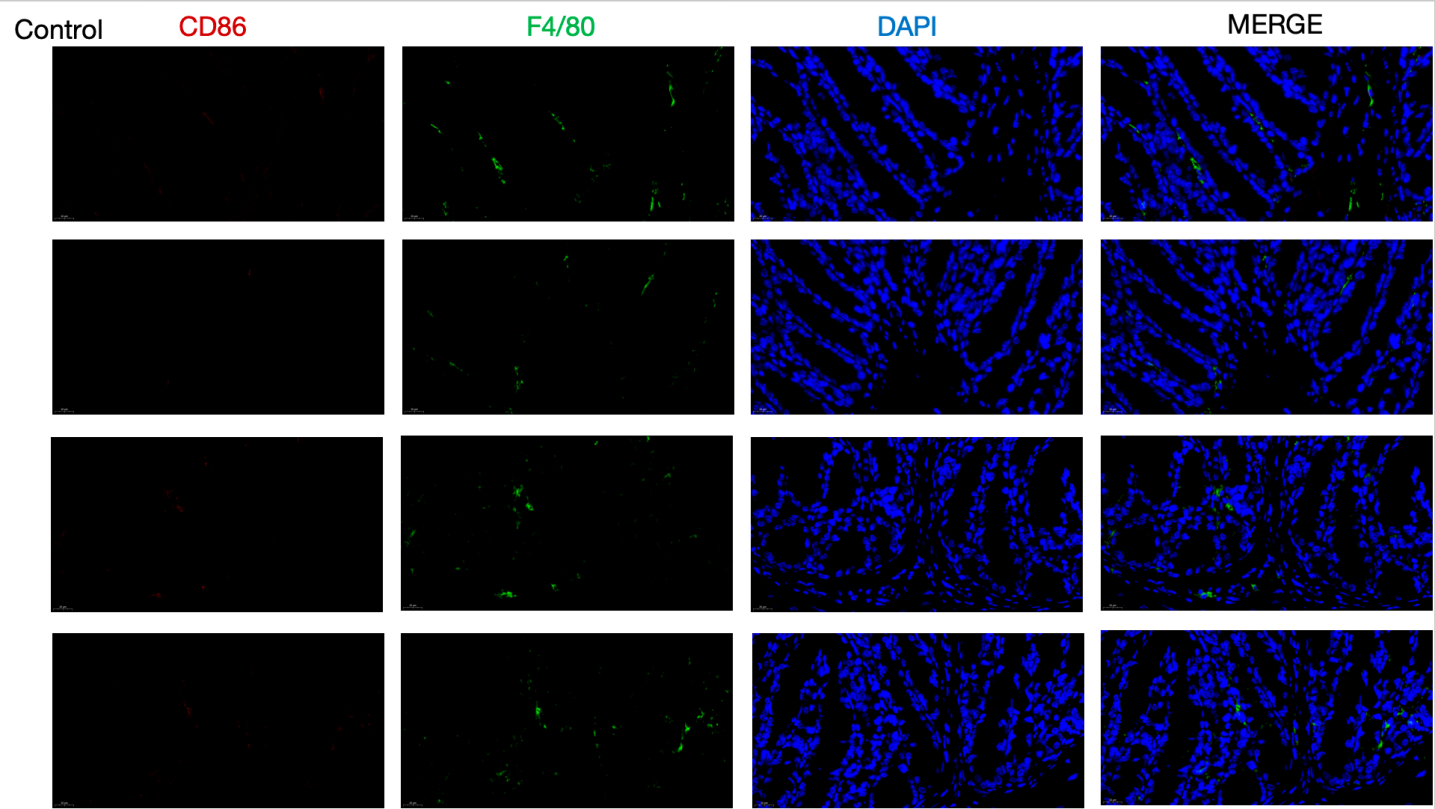


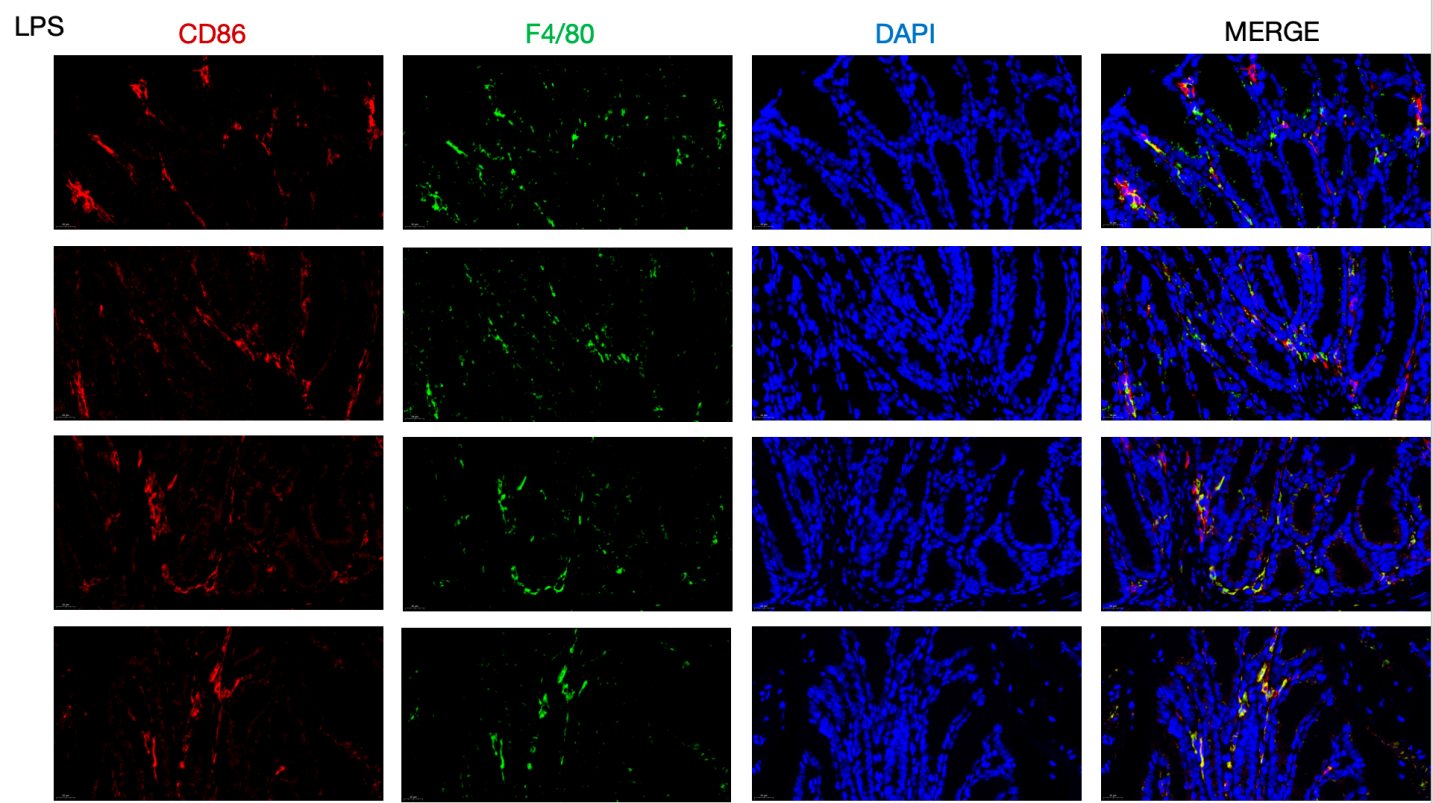


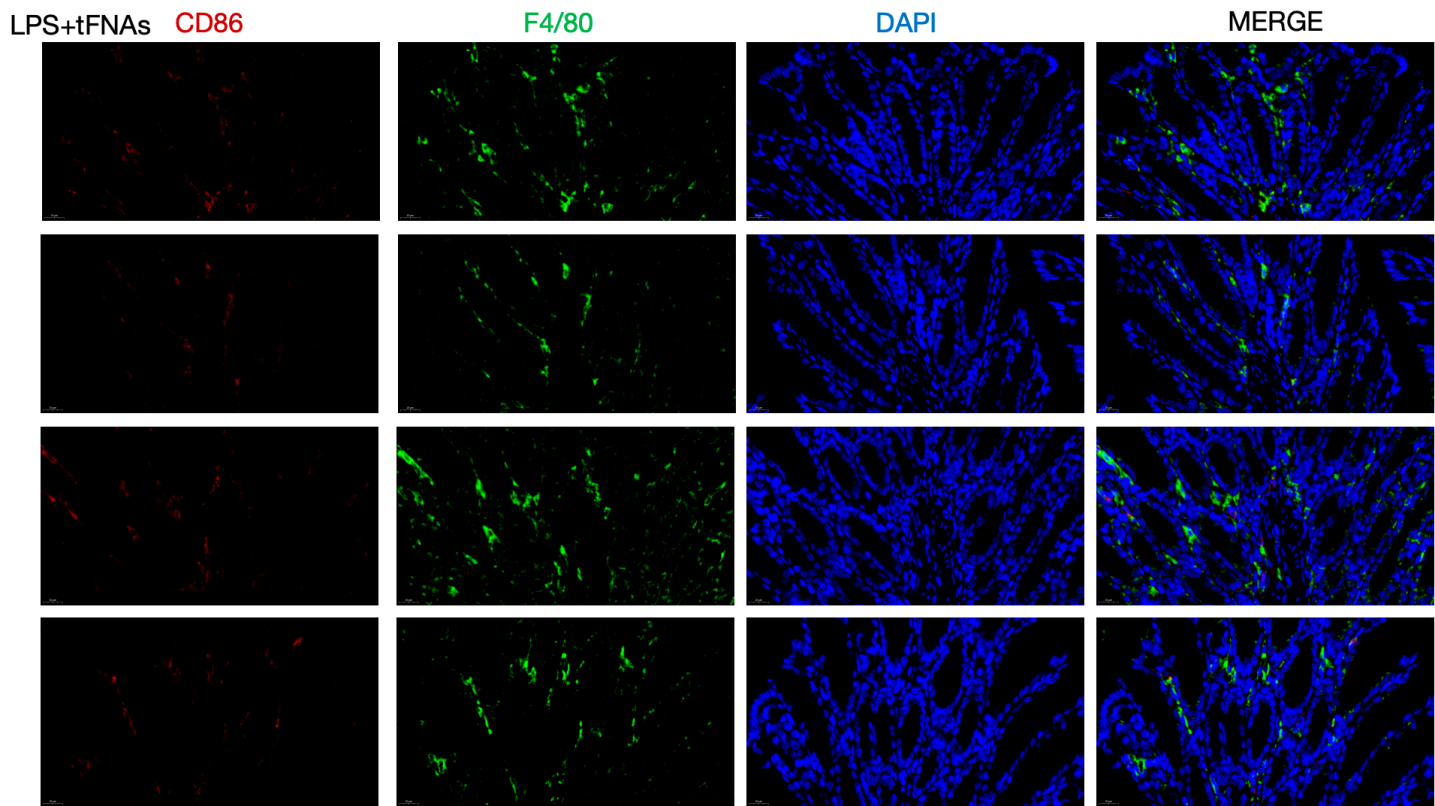


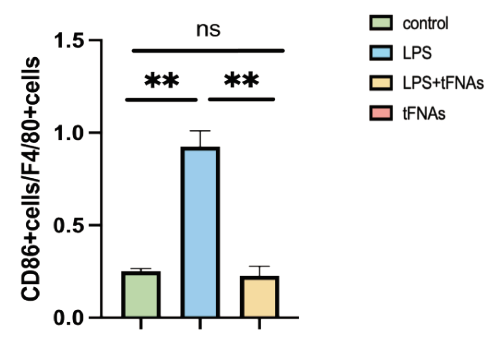


Figure S2. Representative images of immunofluorescent staining for CD86 (red), F4/80 (green), and DAPI (blue) in the colon. Scale bar=50μm. Quantification showing the percentage of CD86 and F4/80 double-positive cells. Data were represented as mean ± SD, *p<0.05, **p<0.01.


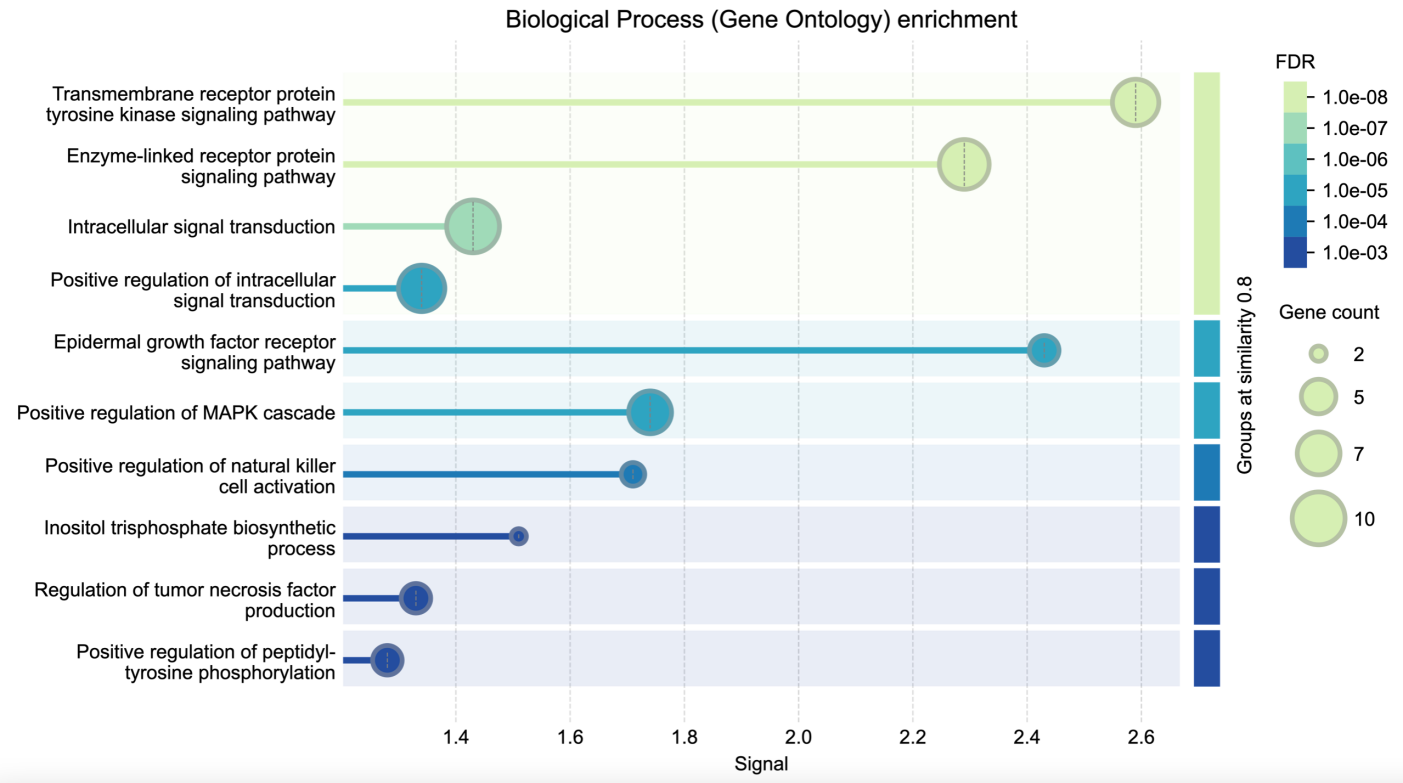


Figure S3.Functional enrichment of Axl gene.Quoted from the STRING website.


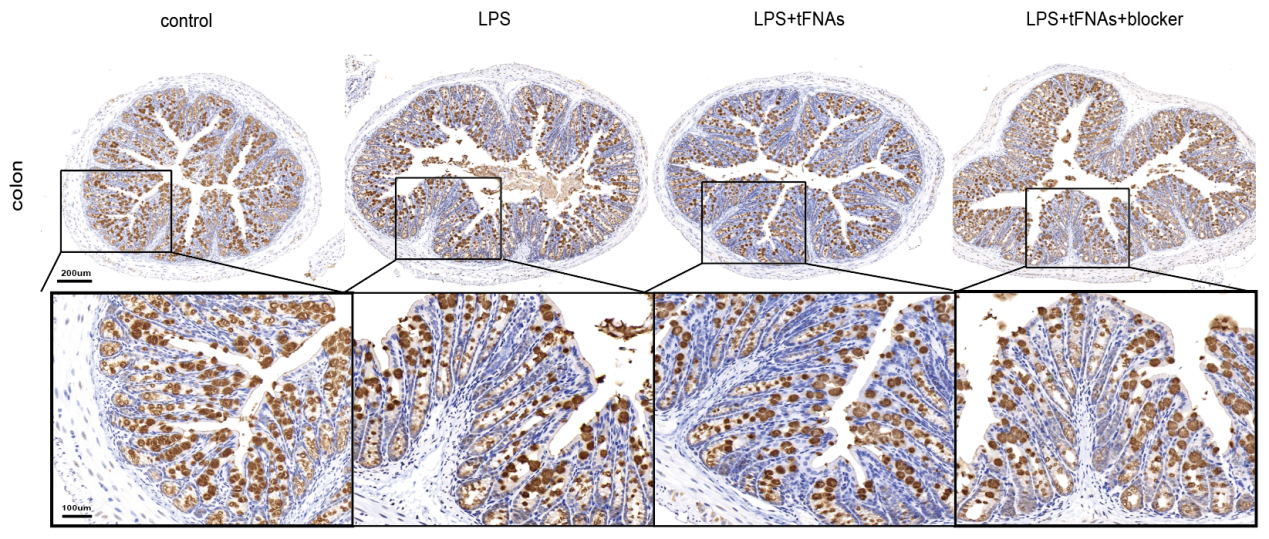


Figure S4. MUC-2 expression in colon (E) revealed by immunohistochemical staining (IHC). (scale bar: 200 μm, scale bar: 100 μm).


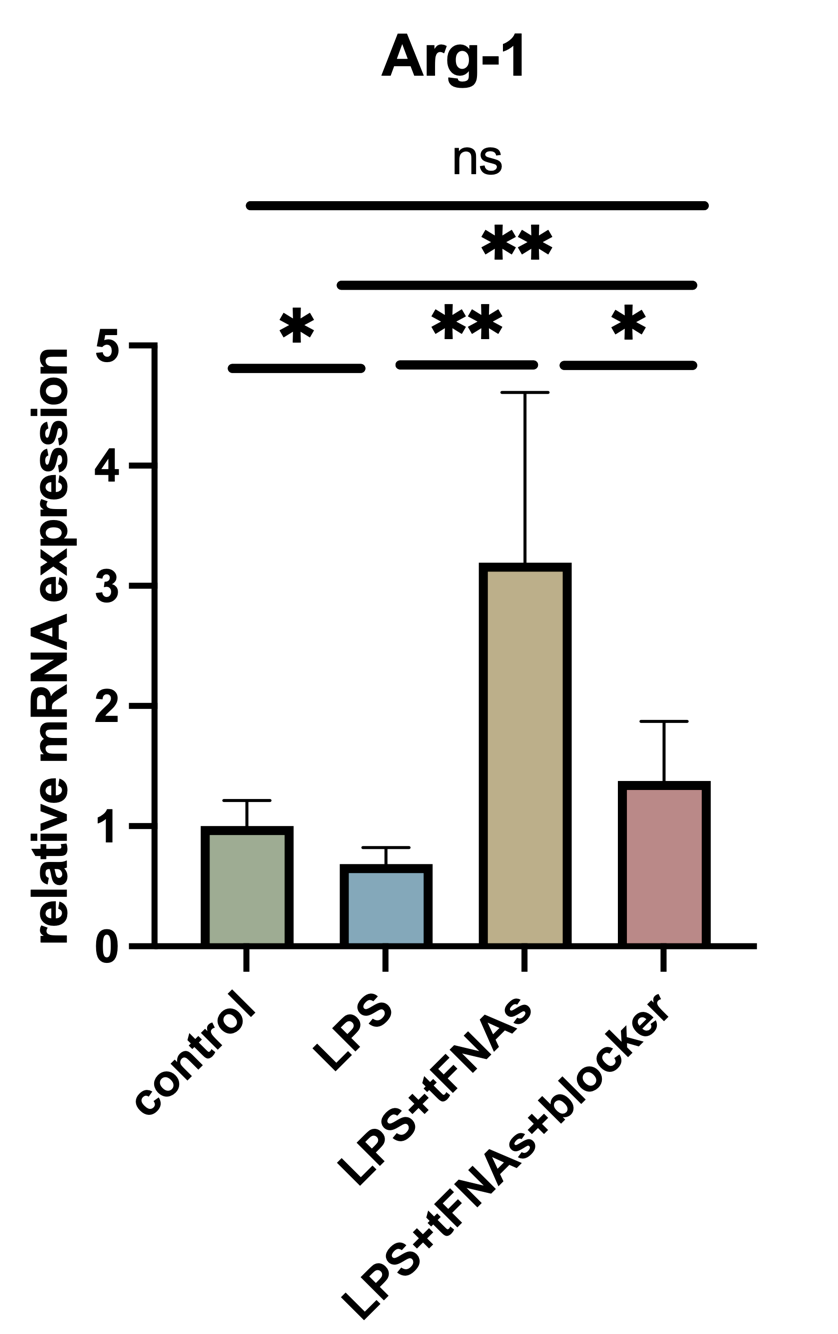


Figure S5. The mRNA level of Arg-1 in colon. Data were analyzed using Student’s t test, Data were represented as mean ± SD, **p*<0.05, ***p*<0.01.


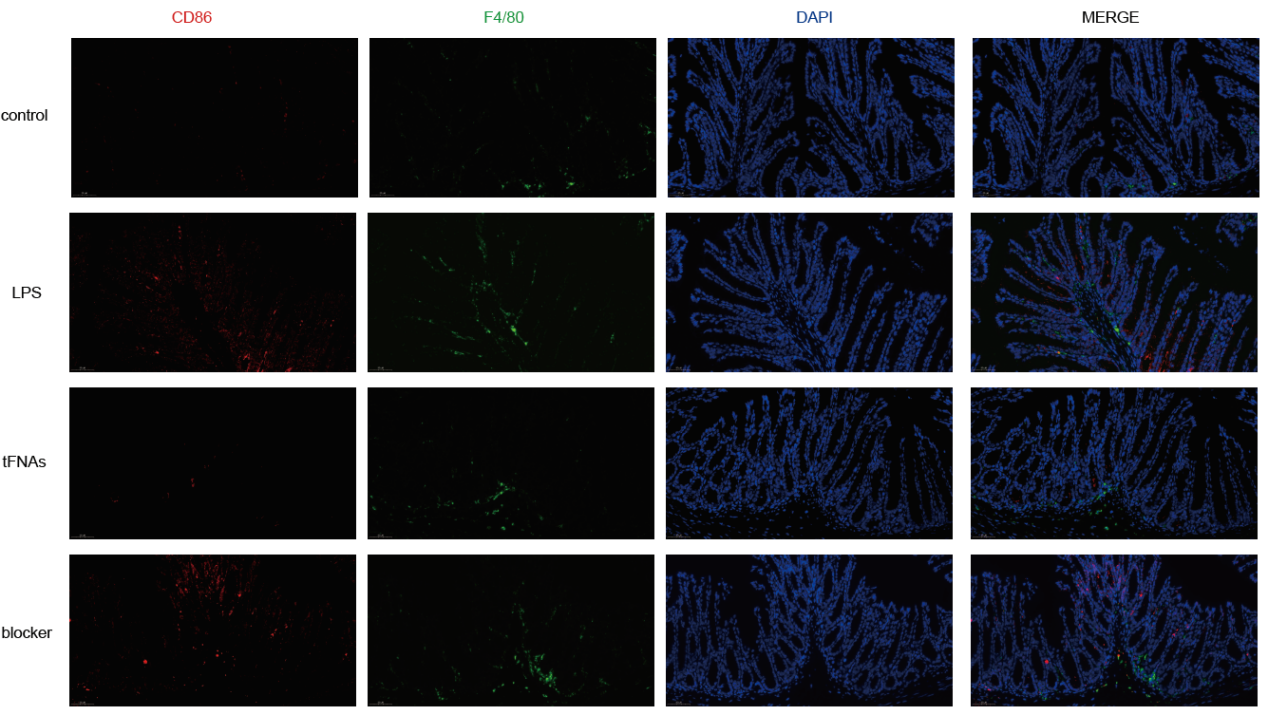


Figure S6.Representative images of immunofluorescent staining for CD86 (red), F4/80 (green), and DAPI (blue) in the colon. Scale bar=50μm.


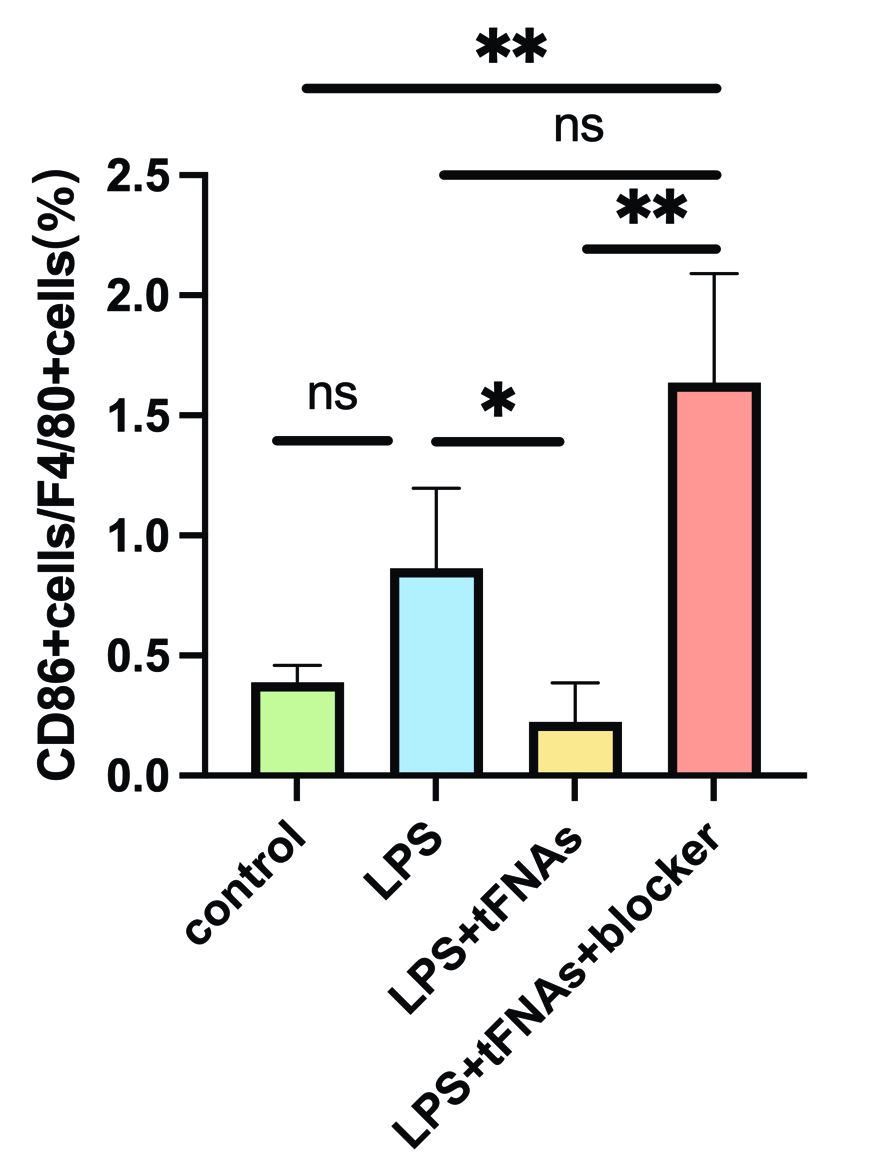


Figure S7. Quantification showing the percentage of CD86 and F4/80 double-positive cells. Data were represented as mean ± SD, **p*<0.05, ***p*<0.01.
